# Topical Application of *Liriope platyphylla* Extract Attenuates Dry Eye Syndrome Induced by Particulate Matter

**DOI:** 10.1155/2019/1429548

**Published:** 2019-12-14

**Authors:** Su Jeong Song, Soo-Wang Hyun, Tae Gu Lee, Bongkyun Park, Kyuhyung Jo, Ik Soo Lee, Chan-Sik Kim

**Affiliations:** ^1^Herbal Medicine Research Division, Korea Institute of Oriental Medicine, Daejeon 34054, Republic of Korea; ^2^Clinical Medicine Division, Korea Institute of Oriental Medicine, Daejeon 34054, Republic of Korea; ^3^Non-clinical Research Collaboration Division, Korea Institute of Oriental Medicine, Daejeon 34054, Republic of Korea; ^4^Korean Convergence Medicine, University of Science and Technology (UST), Daejeon 34054, Republic of Korea

## Abstract

Particulate matter (PM) is a type of air pollutant that poses a risk to human health. In the ocular system, PM causes or aggravates dry eye syndrome (DES) by damaging the corneal and conjunctival epithelia. *Liriope platyphylla* has been used traditionally as an expectorant, antitussive agent, and tonic in Korea. However, the effects of *Liriope platyphylla* extract (LPE) on PM-induced ocular damage have not been elucidated. In this study, we evaluated the *in vivo* protective effect of LPE against PM-induced DES in rats. Topical administration of LPE attenuated the PM-induced decrease in tear volume and reduced corneal epithelial irregularity and damage. LPE also protected against PM-induced disruption of the corneal mucin-4 layer and reduction in the conjunctival goblet cell density. These findings suggest that LPE has protective effects against PM-induced DES.

## 1. Introduction

Particulate matter (PM), a type of environmental air pollution, is a mixture of solid and liquid particles such as sulfite, nitrogen oxide, carbon oxide, heavy metals, benzopyrene, polycyclic aromatic hydrocarbons (PAHs), nitro-substituted PAHs (nitro-PAHs), polychlorinated biphenyl (PCB) congeners, and chlorinated pesticides [1]. PM can penetrate human organs, and it poses a health risk. In the skin, PM exposure increases reactive oxygen species (ROS), epidermal thickening, and dermal inflammation with neutrophil infiltration [2]. Extended PM exposure significantly contributes to the burden of cardiovascular events [3]. Additionally, PM exposure contributes to increased risk and mortality burden of lung cancer [4, 5] and chronic kidney disease [6].

The eyes are another organ damaged by PM exposure. Continued and direct PM exposure may cause various eye symptoms or diseases [7], including allergic conjunctivitis during the nonpollen season [8]; recent studies have found a correlation between dry eye syndrome (DES) and PM exposure [9, 10]. DES affects tears and the ocular surface, resulting in discomfort, tear film instability, and visual disturbances that decrease the quality of life for many patients [11, 12].

DES is a common eye disease, and the number of cases has been increasing around the world. In the United States, 6.8% of adults were diagnosed with DES (∼16.4 million people) in 2017 [13]. Young adults (18 to 45 years) have a 2.7% prevalence, while the rates among adults over 40 years can be as high as 75% [14].

DES can be caused by various factors such as aging, smoking, health condition, and medications, but it can also be caused by PM exposure. Continuous PM exposure reduces tear film stability, influences the tear osmolality [9], delays corneal epithelium wound healing by inhibiting cell migration [15], and increases oxidation stress on the ocular surface [16]. Furthermore, PAH mixtures increase eye irritation and photosensitivity [17], and high concentrations of heavy metals are associated with DES induction [17, 18]. Therefore, PM exposure can induce multifactorial damage to the eyes via the inflammation response and oxidation stress, which increases the possibility of causing or worsening DES.

*Liriope platyphylla* (LP) is a perennial herbaceous evergreen belonging to the lily family. It is cultivated for medicinal or ornamental purposes in Korea, China, Japan, Europe, etc. LP has been used traditionally as an expectorant, antitussive agent, and tonic in Korea [19]. The *Liriope platyphylla* extract (LPE) contains bioactive compounds such as spicatoside A, pennogenin, homo-isoflavonoid, and sitosterol [20, 21].

LP has recently shown clinical effects on diabetes, neurodegenerative disorders, and obesity [22–24]. The root of LP affects hepatitis B viral gene expression and viral DNA replication by nuclear factor (NF-κB) inhibition [25]. Mixtures containing LP have an ability to increase the number of goblet cells and thicken the mucosal layer and muscle fibers [26]. Additionally, LP has shown anti-inflammatory, antioxidant, and wound healing abilities [27, 28]. However, LPE effects have not been well defined in the DES context. Therefore, this study aimed to evaluate the ocular protective effects of LPE on PM-induced DES.

## 2. Materials and Methods

### 2.1. Preparation of LPE

LP (5 kg) was ground, dried, and boiled with 40 L distilled water at 100°C for 3 h. Thereafter, aqueous LPE was concentrated by freeze-drying into powdered form. The concentrated LPE powder was deposited in the herbarium of the Korea Institute of Oriental Medicine (Daejeon, Republic of Korea).

### 2.2. Animals and Experiment Design

**Figure 1:**
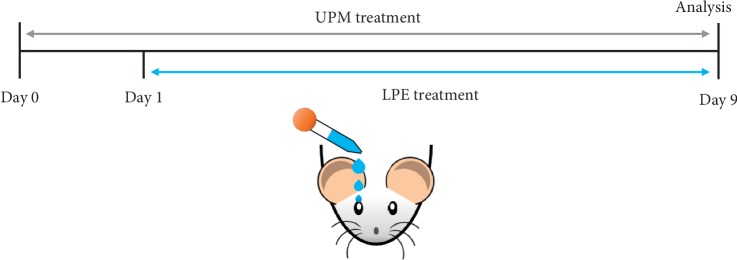
Schematic illustration of animal experiment design. UPM, urban particulate matter; LPE, *Liriope platyphylla* extract.

The animal experiments were conducted in accordance with the Institutional Animal Care and Use Committee approved protocol (IACUC approval ID: 17-060). Six-week-old female Sprague Dawley (SD) rats were purchased from Orient Bio (Seongnam, Korea). Rats were maintained in specific pathogen-free facilities with temperature 22–24°C, relative humidity 50–60%, and 12 h light/12 h dark cycles by the Korea Institute of Oriental Medicine. Urban particulate matter (UPM) was purchased from Sigma-Aldrich (St. Louis, MO, USA; NIST SRM 1648a) and mixed with saline at 20 mg/mL. SD rats were acclimated for 1 week and separated into five groups: (1) Control (CTL); (2) UPM Vehicle group (Veh); (3) UPM with 1 mg/mL LPE; (4) UPM with 5 mg/mL LPE; and (5) UPM with 10 mg/mL LPE). The schematic illustration of the animal experiment is shown in Figure 1. On day 0 through 9, the UPM solution was administered three times via an eyedropper to induce DES. On days 1 through 9, the LPE solution was administered three times via eyedropper after each UPM treatment. On the final day, the UPM and LPE treatment was done only once.

### 2.3. Tear Volume

Tear volume was measured using the phenol red thread (Tianjin Jingming New Technological Development, Tianjin, China). SD rats were anesthetized by intraperitoneal injection of 40 mg/kg pentobarbital (Entobar, Hanlim pharm. Co., LTD, Seoul, Korea). Threads were placed into the third point from the lateral canthus of the lower eyelid for 1 min. The length of the red part was measured using a microscope (SZ61, Olympus, Tokyo, Japan) and presented as the tear volume in millimeters.

### 2.4. Corneal Irregularity Score

The corneal irregularity score was analyzed by the reflection of a ring-shaped slit light on the eye surface using a stereoscopic microscope (SZ61, Olympus, Tokyo, Japan). The corneal irregularity was defined using the following scale: 0, no distortion; 1, distortion in one quadrant; 2, distortion in two quadrants; 3, distortion in three quadrants; 4, distortion in all four quadrants; and 5, severe distortion in which no ring was visible.

### 2.5. Terminal Deoxynucleotidyl Transferases dUTP Nick-End Labeling (TUNEL) Assay

Terminal deoxynucleotidyl transferases dUTP nick-end labeling (TUNEL) was applied to identify corneal cell apoptosis. At necropsy process, the eyes were removed from rats and fixed using 10% formalin solution for 24 h at room temperature. Fixed tissues were dehydrated in ethanol, cleared in xylene, and embedded in paraffin. The paraffin-embedded eye tissues were sectioned using a microtome (Leica, Wetzlar, Germany). Prepared paraffin sections were deparaffinized and stained using the DeadEnd apoptosis detection system (Promega, Madison, WI, USA) according to the manufacturer's protocols. Stained corneal sections were observed using a fluorescence microscope (Olympus). Images of corneal sections were quantified by counting the TUNEL-positive cells.

### 2.6. Immunohistochemistry of Mucin 4 (MUC4)

Corneal paraffin sections were deparaffinized and blocked using the CAS-Block™ Histochemical Reagent (Thermo, Waltham, MA, USA). Sections were washed with PBS and incubated overnight at 4°C with Mucin 4 (MUC4) (Thermo). After incubation, the sections were washed using PBS, marked with an LSAB kit (DAKO, Santa Clara, CA, USA) and specified with a DAB substrate kit (DAKO). The nuclei were counterstained using Hematoxylin qs (VECTOR LABORATORIES, INC., Burlingame, CA, USA). MUC4 levels were analyzed by examining the immunoreactive intensity per unit area (mm^2^) using ImageJ software from NIH (National Institutes of Health, Bethesda, MD, USA).

### 2.7. Periodic Acid-Schiff (PAS) Staining

To confirm the number of goblet cells, paraffin sections of the conjunctiva tissue were stained using the Periodic acid-Schiff (PAS) staining system (Merck, Kenilworth, NJ, USA). After staining, the samples were observed using a DP80 digital camera (Olympus), and the resulting goblet cell images were analyzed using ImageJ software.

### 2.8. Statistical Analysis

Statistical analysis was performed using one-way analysis of variance (ANOVA) followed by Tukey's multiple comparison test between groups using the Prism 7.0 Software (GraphPad, San Diego, CA, USA). The data from the fluorescein staining score and irregularity score were analyzed using the Kruskal–Wallis nonparametric ANOVA with Dunn's multiple comparisons test; *p* values <0.05 indicated statistical significance.

## 3. Results

### 3.1. LPE Recovers Tear Secretion

**Figure 2:**
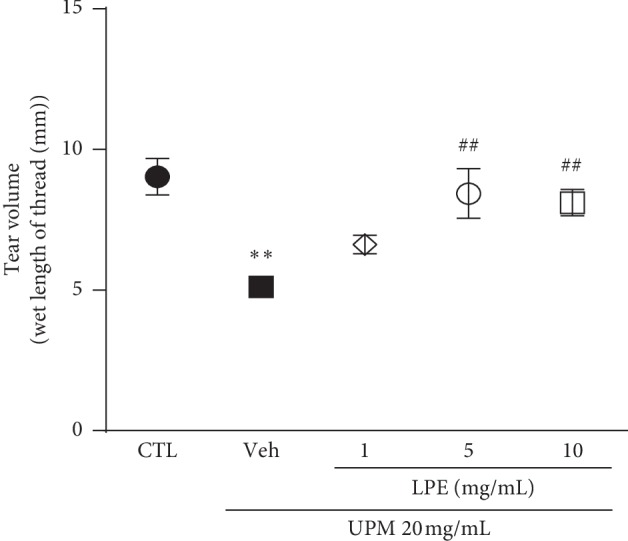
Tear volume analysis of urban particulate matter (UPM)-induced dry eye syndrome (DES) with and without *Liriope platyphylla* extract (LPE) treatment (1, 5, and 10 mg/mL). CTL, Control; Veh, Vehicle. Data shown are mean ± standard error. ^*∗∗*^*p* < 0.01 vs. Control; ^##^*p* < 0.01 vs. Vehicle.

To assess the degree of UPM-induced DES in SD rats, we measured tear volume with and without LPE (Figure 2). The UPM-treated group (9.0 ± 0.65 mm) showed considerably reduced tear volume compared to that of the control group (5.1 ± 0.32 mm). The tear volume was significantly recovered in the 5 and 10 mg/mL LPE-treated groups, respectively, and was similar to that of the control group (8.4 ± 0.88 and 8.1 ± 0.47 mm, respectively).

### 3.2. LPE Effects on Corneal Smoothness

**Figure 3:**
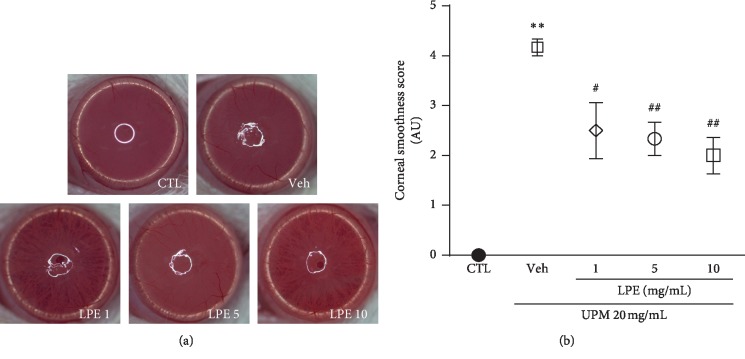
The effect of *Liriope platyphylla* extract (LPE) on corneal smoothness in urban particulate matter (UPM)-induced dry eye syndrome (DES). (a) Representative corneal images of Control (CTL), Vehicle (Veh), and LPE (1, 5, and 10 mg/mL) groups. (b) Graph of corneal irregularity scores. Data shown are mean ± standard error. ^*∗∗*^*p* < 0.01 vs. CTL; ^#^*p* < 0.05 or ^##^*p* < 0.01 vs. Veh.

The corneal irregularity score is an index of the corneal surface smoothness and damage (Figure 3). The corneal irregularity score of the UPM-treated group (4.2 ± 0.17 AU) increased compared to the control group. The corneal irregularity scores in the 1, 5, and 10 mg/mL LPE-treated groups were lower than those in the Veh group (2.5 ± 0.56, 2.3 ± 0.33, and 2.0 ± 0.37 AU, respectively).

### 3.3. LPE Protects the Corneal Surface

**Figure 4:**
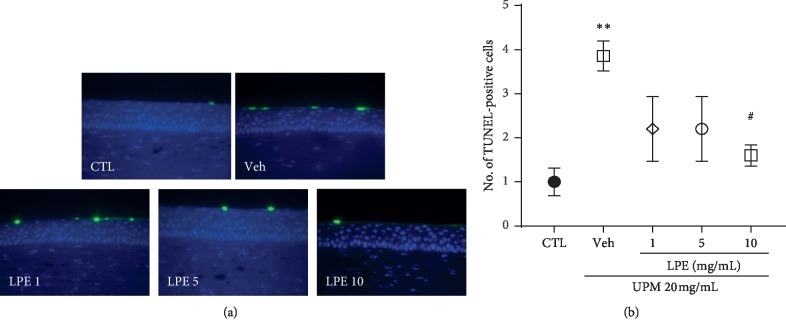
Terminal deoxynucleotidyl transferases dUTP nick-end labeling (TUNEL) assay. (a) Representative images of TUNEL-positive cells within the Control (CTL), Vehicle (Veh), and *Liriope platyphylla* extract (LPE) (1, 5, and 10 mg/mL) groups. (b) Graph of the number of TUNEL-positive cells. Data shown are mean ± standard error. ^*∗∗*^*p* < 0.01 vs. CTL; ^#^*p* < 0.05 vs. Veh.

As shown in Figure 4(a), the UPM-treated group showed a stronger apoptotic signal on the ocular surface than the CTL group. The 10 mg/mL LPE group showed a weaker apoptotic signal than the UPM-treated group. For a detailed assay, the number of TUNEL-positive cells was counted in areas of the same size (Figure 4(b)). The UPM-treated group (3.9 ± 0.34) showed more TUNEL-positive cells than the CTL group (1.0 ± 0.32). The 10 mg/mL LPE group (1.6 ± 0.24) showed significantly lesser TUNEL-positive cells than the UPM-treated group.

### 3.4. LPE Recovers the Level of MUC4

**Figure 5:**
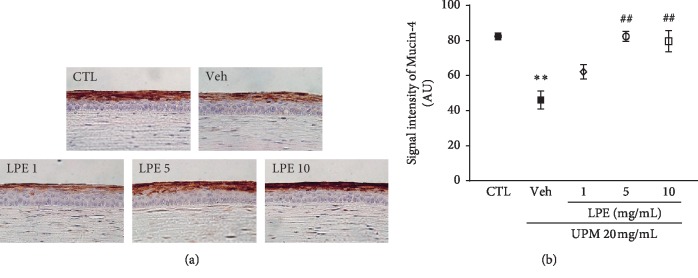
The effect of *Liriope platyphylla* extract (LPE) on a mucin protein (MUC4) in urban particulate matter (UPM)-induced dry eye syndrome (DES). (a) Representative images of MUC4-stained corneal sections of the Control (CTL), Vehicle (Veh), and LPE (1, 5, and, 10 mg/mL) groups. (b) Graph of MUC4 signal intensity. Data shown are mean ± standard error. ^*∗∗*^*p* < 0.01 vs. CTL; ^##^*p* < 0.01 vs. Veh.

Figure 5(a) shows images of MUC4-stained corneal sections. The Veh group had lower MUC4 levels than the CTL group. The LPE-treated group presented a gradual, dose-dependent increase in the MUC4 layer. We quantified the signal intensity of the MUC4 layer for a detailed assay (Figure 5(b)). The signal intensity of MUC4 in the UPM-treated group (46.0 ± 5.20 AU) was decreased compared to that of the CTL group (82.4 ± 1.88 AU). The 1 mg/mL LPE-treated group showed a slightly increased MUC4 signal compared to that of the Veh group. The 5 and 10 mg/mL LPE-treated groups (82.3 ± 2.90 and 79.5 ± 6.04 AU, respectively) had enhanced MUC4 intensity, similar to the CTL group.

### 3.5. LPE Restores Conjunctival Goblet Cells

**Figure 6:**
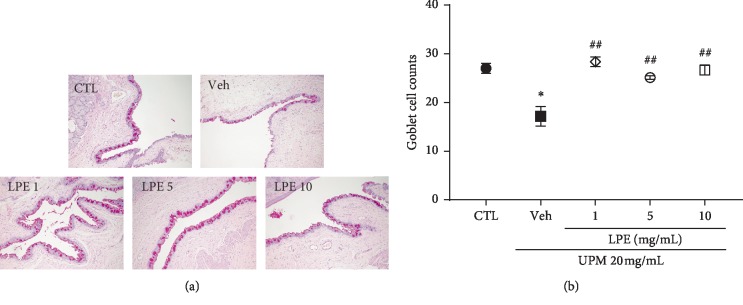
Effect of *Liriope platyphylla* extract (LPE) on conjunctival goblet cells in urban particulate matter (UPM)-induced dry eye syndrome (DES). (a) Representative images of goblet cells stained with periodic acid-Schiff (PAS). (b) Graph of goblet cell counts. Data shown are mean ± standard error. ^*∗*^*p* < 0.05 vs. Control (CTL); ^##^*p* < 0.01 vs. Vehicle (Veh).

As shown in Figure 6, the UPM-treated group (17.2 ± 2.00) showed fewer goblet cells than the CTL group (27.0 ± 1.00). The number of goblet cells recovered in the 1, 5, and 10 mg/mL LPE-treated groups was 28.4 ± 1.00, 25.1 ± 0.40, and 26.7 ± 1.03, respectively.

## 4. Discussion

Accelerated industrialization and urbanization have been generating increased levels of environmental pollutants such as PM [29]. Although several studies have addressed the effects of PM on the development of human cardiovascular [3] and respiratory diseases [30], our current study focused on eye disease because the eyes are in direct contact with PM. Several recent studies have reported that PM causes or aggravates DES [3, 31]. We applied UPM from NIST as a standard reference PM to induce DES because the UPM constituents (such as polycyclic aromatic hydrocarbons (PAHs), nitro-substituted PAHs (nitro-PAHs), polychlorinated biphenyl (PCB) congeners, and chlorinated pesticides) are similar to the atmospheric PM within industrialized urban areas.

DES is driven by low lacrimal flow and high evaporation that leads to reduced tear volume [32]. The exposure to PM also induces significant toxicity and damage to the ocular surface, including the cornea and conjunctiva [16]. Damaged corneal and conjunctival epithelia lead to barrier dysfunction, which is ascribed to dry eye syndrome [33]. Epithelial damage is mainly caused by cell death, including apoptosis. It has been reported that PM may directly cause corneal epithelial apoptosis [16]. In this study, we observed that PM decreased tear volume and LPE recovered the decreased tear volume due to PM. LPE also prevented the increase in corneal irregularity scores and decreased apoptosis induced by PM. These results show that LPE may relieve DES symptoms and protect the ocular surface from the damage caused by PM exposure.

Damage to the corneal and conjunctival epithelia also involves the disturbance of mucin expression and loss of goblet cells [34]. Mucins are high-molecular weight glycoproteins that are expressed on the ocular epithelial surface [35]. They are classified into three categories based on their amino acid sequences: transmembrane (MUC1, MUC4, MUC16, etc.) and gel-forming (MUC5AC, etc.), soluble (MUC7 and MUC9), and unclassified (MUC8 and MUC11) types [36].

Ocular surface membrane-associated mucins are found in the mucin layer of the corneal and conjunctival epithelia; the gel-forming mucins are mainly found in the water layer. Mucins play a role in keeping the ocular surface wet [36] and protecting it from pathogens, allergens, and extracellular molecules [37]. The gel-forming mucins are produced by goblet cells in the conjunctival epithelium. Goblet cells maintain homeostasis within the conjunctival epithelium and are important in DES development.

The alteration of ocular mucins is also related to DES progression [38]. In addition, it is reported that PM exposure impacts the corneal MUC4 layer [31] and the density of conjunctival goblet cells [39]. In this study, LPE thickened the MUC4 layer and increased the conjunctival goblet cell density under PM exposure. These results revealed that LPE may have an ability to protect and recover the disruption of mucin induced by PM.

LPE includes steroid saponins (such as spicatoside A) and flavonoids [20, 21]. Among the bioactive compounds, spicatoside A is reported to have antioxidative and anti-inflammatory effects [40–42] and can induce an increase in the production and secretion of mucins [43]. In addition, the combination of various flavonoids and saponins is expected to provide eye protection. Further experiments will aim to demonstrate the mechanisms behind the effects of individual bioactive compounds and the LPE-induced mucin upregulation under PM exposure.

## 5. Conclusion

In conclusion, our results show that LPE treatment improved DES, which was confirmed based on changes in tear volume, corneal irregularity, apoptosis, MUC4 levels, and goblet cell numbers. The results suggest that LPE has the potential to relieve DES through its ocular protective effects.

## Data Availability

All data and materials used are described in the article. Upon request, the corresponding author can provide further information.
